# Impact of Concurrent Media Exposure on Professional Identity: Cross-Sectional Study of 1087 Medical Students During Long COVID

**DOI:** 10.2196/50057

**Published:** 2024-10-17

**Authors:** Manli Wu, Jun Yan, Chongming Qiao, Chu Yan

**Affiliations:** 1 School of Journalism and Information Communication Huazhong University of Science and Technology Wuhan China; 2 School of Media and Communication Shanghai Jiao Tong University Shanghai China; 3 School of Marxism Huazhong University of Science and Technology Wuhan China

**Keywords:** COVID-19, media exposure, social support, professional identity, medical students, Stimulus-Organism-Response framework

## Abstract

**Background:**

Long COVID has widened the health gap across society and highlighted the vulnerabilities and risks faced by health care systems. For instance, the global trend of medical workers resigning has become a prominent topic on social media. In response to this severe social problem in global public health within the digital society, it is urgent to investigate how the professional identity of medical students, who are digital natives and the future workforce of medical practitioners, is affected by the media environment.

**Objective:**

This study aims to examine how media exposure relates to medical students’ perceptions of informational and emotional support, and how these perceptions further influence the development of their professional identity.

**Methods:**

Building on the Stimulus-Organism-Response (SOR) framework, this study develops a theoretical model to illustrate how media exposure affects medical students’ professional identity through the mediation of social support. Specifically, media exposure was assessed through online news media and social media exposure; social support was evaluated in terms of informational and emotional support; and professional identity was measured through medical students’ sense of belonging and professional commitment. A survey was conducted at a medical school in China, yielding 1087 valid responses that were analyzed using SmartPLS 4.0.

**Results:**

Consistent with our expectations, online news media exposure was positively associated with both informational support (β=.163; *P*<.001) and emotional support (β=.084; *P*=.007). Similarly, social media exposure showed positive associations with informational support (β=.122; *P*<.001) and emotional support (β=.235; *P*<.001). Thereafter, informational support (β=.228; *P*<.001) and emotional support (β=.344; *P*<.001) were positively associated with students’ sense of belonging. Meanwhile, both informational support (β=.245; *P*<.001) and emotional support (β=.412; *P*<.001) positively impacted medical students’ professional commitment. In addition, a mediation test was conducted. The results confirmed that informational support and emotional support partially mediated the effect of online news media, while fully mediating the effect of social media on medical students’ sense of belonging and professional commitment.

**Conclusions:**

This study finds that exposure to online news media and social media can enhance medical students’ sense of belonging and professional commitment through the formation of informational and emotional support. It expands the discussion on the role of media in providing social support and facilitating the development of medical students’ professional identity. This is a valuable contribution to addressing complex public health crises through effective media governance in the network era.

## Introduction

### Background

Media factors in medical sociology have long been recognized as significant in influencing health management and medical outcomes. For example, media and media exposure have been shown to play a key role in heart disease prevention, vaccination, community care, and other health interventions [1,2]. The COVID-19 pandemic and long COVID in the digital age have caused unprecedented social disruptions due to the widespread use of social media and the circulation of mixed information. Therefore, it is essential to investigate how these new “media factors” impact the stability and development of society.

Furthermore, the prevalence of COVID-19 is accompanied by an infodemic [3]. The disaster stories about COVID-19 on social media serve not only as a significant force to unite the public in combating the pandemic but also as hidden triggers for the widespread resignation of health care workers. According to the World Health Organization (WHO), there will be a shortfall of 10 million health workers by 2030 [4]. Medical students are a vital reserve force for health care workers and play an essential role in public health events. For instance, during the COVID-19 pandemic, medical students’ involvement in patient care and health consultation complemented the work of medical professionals [5,6]. Their professional identity is critical to their career perception and choices [7], which may further impact the stability of the medical workforce.

Professional identity refers to the self-conceptualization of one’s work role [8] and is central to professional loyalty. In contrast to experienced medical professionals, medical students are young, vulnerable, and digital natives, with their professional identities still in formation and transformation [9]. Prior studies have typically applied descriptive analyses to explain the development of professional identity [10,11]. Although current studies are beginning to use cross-sectional methods to explore predictors of professional identity, they primarily discuss individual and educational environmental factors, such as clinical practice [12], experiences within the educational system [13], and working conditions [14]. Recently, there has been growing evidence suggesting that online media play an important role in influencing students’ knowledge and attitudes regarding the medical profession [15]; however, the theoretical mechanisms behind this influence remain unclear. Given that the COVID-19 pandemic has highlighted the impact of media on people’s daily lives [16], this research speculates that exposure to online media during the pandemic could also influence medical students’ professional identity. As online news media and social media are the most commonly used types of media, this study proposes the following research question: *How does concurrent media exposure affect medical students’ professional identity?*

In public health events such as the COVID-19 pandemic, the media play a crucial role in information distribution, pandemic warnings, gauging public emotions, facilitating social mobilization, and providing emotional support [17]. Specifically, online media not only deliver medical-related information [18] but also feature news reports that portray the professional and heroic images of health care workers [19]. Additionally, online media facilitate social interactions [20], allowing netizens to express their support for health care workers through understanding, encouragement, affirmation, sympathy, and care, among other forms [21]. As such, the social support gained through online media exposure may help medical students recognize the value of medical groups [22], develop their attitudes toward the medical profession, and understand public opinions about the field [23]. This study utilizes the Stimulus-Organism-Response (SOR) framework [24] to explain how media exposure relates to medical students’ professional identity, emphasizing the mediating role of social support.

To address the proposed research question, a survey method is used, yielding a total of 1087 valid responses. The results reveal that media exposure during the COVID-19 pandemic affects medical students’ professional identity, either directly or indirectly, through social support. Theoretically, this study provides a comprehensive understanding of the effects of media exposure on the development of medical students’ professional identity by extending the SOR framework. Practically, this study underscores the importance of media and information management in fostering professional identity, offering a viable approach to addressing the shortage of the medical workforce, promoting social unity, and reducing health disparities.

### Objectives

The objective of this study was to examine the effect of concurrent media exposure on medical students’ professional identity, with a specific focus on the mediating role of social support. In particular, this study investigated how concurrent media exposure (ie, online news media and social media) influenced social support (ie, informational support and emotional support), which in turn affected the development of medical students’ professional identity (ie, sense of belonging and professional commitment).

### Theoretical Background

#### Professional Identity

As a crucial link between individuals and society, professional identity is an essential force in fostering social unity and mitigating social risks. Professional identity encompasses the attitudes, values, knowledge, beliefs, and skills shared among members of a professional group [8]. It not only influences individuals’ career choices [25] but also impacts their job satisfaction [26] and turnover intentions [27]. Prior studies have typically focused on specific professions, such as teachers, lawyers, and health care workers [26,28,29]. Professional identity is especially significant in medical education, as it can influence the development of the medical workforce [25].

Although prior studies have generally examined professional identity as an integrated concept [26,30], it can also be understood as a complex structure [31]. Individuals typically develop their identity based on their feelings and behaviors toward the profession [32]. To gain a nuanced understanding of professional identity, this study uses belonging and professional commitment as its key components. Belonging refers to the feeling of being an integral part of a professional group, encompassing perceptions of acceptance, security, and respect within the specific profession [33]. Professional commitment reflects both attitudes and behaviors toward the profession, indicating the relative strength of an individual’s identification with their profession and their willingness to invest effort in and maintain membership within it [34]. Professional commitment reflects the alignment between personal beliefs and professional goals [35], which can lead individuals to invest effort and develop a sense of attachment to their profession. It comprises 3 dimensions: affective commitment, continuance commitment, and normative commitment. Affective commitment pertains to individuals’ attitudes or feelings toward the profession, while continuance and normative commitment relate to individuals’ behavioral intentions regarding their profession [36].

Belonging and professional commitment represent different aspects of professional identity, and it is important to recognize the distinctions between them. Belonging is a psychological concept that reflects the relationship between an individual and others within a particular group [33,37], while professional commitment measures the intensity of an individual’s connection to their profession, characterized by the willingness to invest effort and the desire to remain in that profession [36]. In this context, belonging emphasizes individuals’ psychological ownership of a group [37], while professional commitment serves as a stabilizing or obliging force that guides one’s professional behavior [38].

#### SOR Framework

##### Overview

Derived from environmental psychology, the SOR framework posits that environmental factors can trigger cognitive and affective reactions in individuals, which subsequently influence their behaviors [24]. The SOR framework consists of 3 interconnected components: stimulus, organism, and response. Specifically, the stimulus refers to the environmental factors that impact individuals’ psychological states [24]. Response refers to behavior in reaction to a certain stimulus [24,39]. The organism, consisting of cognitive and affective states [39], is often considered the mediator between stimulus and response [40,41]. Cognitive states involve the acquisition, processing, retention, and retrieval of information, while affective states include pleasure, arousal, dominance [24], and other emotion typologies relevant to specific contexts [39].

The SOR framework has been widely used to explain individuals’ behaviors. For instance, previous research has used this framework to assess the impacts of social media overload [42] and technological features [43] on users’ behaviors. In a recent study, the SOR framework was applied to examine the impact of the COVID-19 pandemic on behavioral changes among university students [44]. This study adopts the SOR framework for 2 reasons. First, the SOR framework delineates the stages of behavior formation by highlighting how external cues influence individuals’ responses through changes in their internal states [24], thereby illuminating the development of medical students’ professional identity. Second, the organism component encompasses both cognitive and affective reactions [39], allowing us to differentiate medical students’ psychological responses resulting from media exposure.

Along with the rationale behind the SOR framework, this study treats media exposure as an external stimulus. Given that media exposure is a significant source of social support [45,46], this study conceptualizes social support as the organism component, with emotional support representing the affective aspect and informational support reflecting the cognitive aspect. Furthermore, professional identity is viewed as the response of medical students in reaction to media exposure.

##### Media Exposure as Stimulus

Media exposure refers to the frequency with which individuals are exposed to specific media [47]. It has been recognized as a critical predictor of various behaviors, including political participation [48], sexual behavior [49], and alcohol consumption [50]. During the COVID-19 pandemic, people increasingly relied on media to obtain real-time information for making health decisions [51]. Existing literature has validated the effects of media exposure on the public’s preventive behaviors [1]. In general, media exposure can influence individuals’ cognition and behaviors in the age of COVID-19. Through media exposure, medical students can learn about the responsibilities and challenges faced by medical workers [19], which may impact their professional identity.

Among all media categories, online news media and social media are the 2 most representative and frequently used channels in the new media environment [20]. Unlike traditional news media, such as television and newspapers, online news media rely on the internet to deliver news content. As a result of the news gatekeeping mechanisms, content on online news media typically originates from authoritative sources, reflecting official attitudes and the mainstream values of society [20]. Therefore, people often place their trust in online news media. By contrast, anyone can post and share information on social media, and quality control over content is limited [52]. As social media facilitate online interactions, they have become a prominent source of information during COVID-19 [53]. This study simultaneously considers online news media and social media as media stimuli, as they are the primary information sources and may influence medical students differently.

##### Social Support as an Organism

Through online media, social support can be delivered via information transmission and online interactions [53]. Social support refers to the tangible or intangible resources obtained from others through interpersonal ties within a social network [54]. Prior studies have confirmed the importance of social support in coping with stress and depression [55], improving psychological health [56], and enhancing life satisfaction [57]. Social support is available through online media in various forms, including companionship, solidarity, and information exchange [46,58]. Among all categories of social support, informational support and emotional support are the most frequently mentioned [45,59,60]. Informational support emphasizes the provision of information through giving advice, offering appraisal support, sharing new information, or providing references [59,61]. Emotional support, by contrast, refers to the affective assistance characterized by caring, empathy, love, and trust [61]. Although both informational support and emotional support are psychological variables reflecting users’ perceptions of support availability, informational support focuses on problem-solving, while emotional support pertains to affective assistance [62]. Therefore, these 2 dimensions may have differential impacts. Consequently, this study treats social support as the organism component, with emotional support representing the affective aspect and informational support representing the cognitive aspect.

### Hypotheses Development

#### Study Rationale

This study examines how media exposure influences medical students’ professional identity, with a particular focus on the underlying mechanisms. Based on the rationale of the SOR framework, exposure to online news media and social media can impact both informational and emotional support, which, in turn, affects medical students’ professional identity.

#### The Effects of Online News Media Exposure on Social Support

Online news media play a crucial role in providing pandemic-related information. Given that people tend to have a high level of trust in information from authoritative sources [2,20], medical students are more likely to trust and adopt information presented in online news media. This exposure allows medical students to acquire professional knowledge to navigate the pandemic and to learn about the contributions and sacrifices of health care workers [19]. Therefore, exposure to online news media can offer valuable informational support to medical students.

In addition, news content on online news media typically embodies mainstream values and reflects official attitudes [20]. As a result of the dedication of health care workers during the pandemic, medical groups are often highly praised in news reports [19]. Exposure to online news media may help medical students recognize that health care workers are respected and valued by the public. Furthermore, by allowing readers to comment on specific news reports, online news media serve as channels for the public to express their respect for health care workers. Thus, online news media can also provide emotional support to medical students. Therefore, the following hypotheses are proposed:

- H1a: Online news media exposure is positively associated with informational support.
- H1b: Online news media exposure is positively associated with emotional support.

#### The Effects of Social Media Exposure on Social Support

With abundant user-generated content and multiple information sources, social media play an important role in the dissemination of medical information [63]. Through social media, exposure to content related to medical workers not only helps medical students protect their health but also enriches their professional knowledge. Furthermore, as mutual trust can be established through online interactions [20,22], medical students likely place significant value on information shared by individuals within their online social networks.

Moreover, exposure to social media can facilitate emotional communication through social network connections [64]. Users can share personal feelings with one another through online interactions. In doing so, emotional support—such as understanding, encouragement, affirmation, sympathy, or care—is conveyed [21]. During the pandemic, social media enabled medical students to stay in contact with their acquaintances and support one another. Therefore, the following hypotheses are proposed:

- H2a: Social media exposure is positively associated with informational support.
- H2b: Social media exposure is positively associated with emotional support.

#### The Effects of Informational Support on Professional Identity

During the pandemic, information provided by medical professionals not only helped the public but also benefited medical students. Individuals who receive help from others are more likely to develop a positive attitude toward those providing the assistance [14]. Belonging refers to the extent to which individuals integrate themselves into their recognized group [37]. Thus, medical students who receive informational support from medical professionals may come to appreciate the values of medical groups and foster long-term relationships with them. In this way, a sense of belonging is cultivated.

Information about the pandemic can also offer medical students guidelines for future practice and deepen their understanding of medical practitioners’ responsibilities. These perceived messages help foster acceptance of professional norms and recognition of the obligations within the “psychological contract” established with the medical profession, both of which are crucial components of commitment [36,38]. In this context, informational support accelerates the development of medical students’ professional commitment. Therefore, the following hypotheses are proposed:

- H3a: Informational support is positively associated with medical students’ sense of belonging to the medical profession.
- H3b: Informational support is positively associated with medical students’ professional commitment.

#### The Effects of Emotional Support on Professional Identity

Emotional support also plays a key role in shaping medical students’ attitudes toward medical groups and the profession. Previous research has shown that emotional support from colleagues can strengthen an individual’s attachment to the group [60]. Specifically, trust and respect toward medical workers can instill pride in the profession, further enhancing medical students’ identification as part of the medical community [23]. In this way, emotional support can reinforce medical students’ sense of belonging to the medical profession.

Emotional support can also influence professional commitment. For example, it has been shown to reduce burnout among nurses [65] and promote their commitment to the profession [66]. Similarly, emotional support may strengthen medical students’ professional commitment by improving their psychological well-being. According to Ahmad et al [67], a supportive environment can increase student engagement and enhance their commitment to their profession. Emotional support from social networks can help create such an environment, further boosting medical students’ professional commitment. The following hypotheses are proposed:

- H4a: Emotional support is positively associated with medical students’ sense of belonging to the medical profession.
- H4b: Emotional support is positively associated with medical students’ professional commitment.

#### The Mediation Effects of Social Support

Media exposure can influence the development of individuals’ professional identity. Evidence indicates that exposure to media representations related to one’s identity can affect their understanding of social groups [19,68] and shape their identification with a specific identity [69]. Notably, Geusens and Beullens [69] proposed that media might impact the perception of one’s identity in an indirect manner. This study uses social support as a potential explanatory mechanism to clarify the relationship between media exposure and professional identity. Therefore, it hypothesizes that media exposure may indirectly influence medical students’ professional identity through the mediation of social support. The following hypotheses are proposed:

- H5: Informational support mediates the effects of online news media on medical students’ sense of belonging (a) and professional commitment (b).
- H6: Emotional support mediates the effects of online news media on medical students’ sense of belonging (a) and professional commitment (b).
- H7: Informational support mediates the effects of social media on medical students’ sense of belonging (a) and professional commitment (b).
- H8: Emotional support mediates the effects of social media on medical students’ sense of belonging (a) and professional commitment (b).

**Figure 1:**
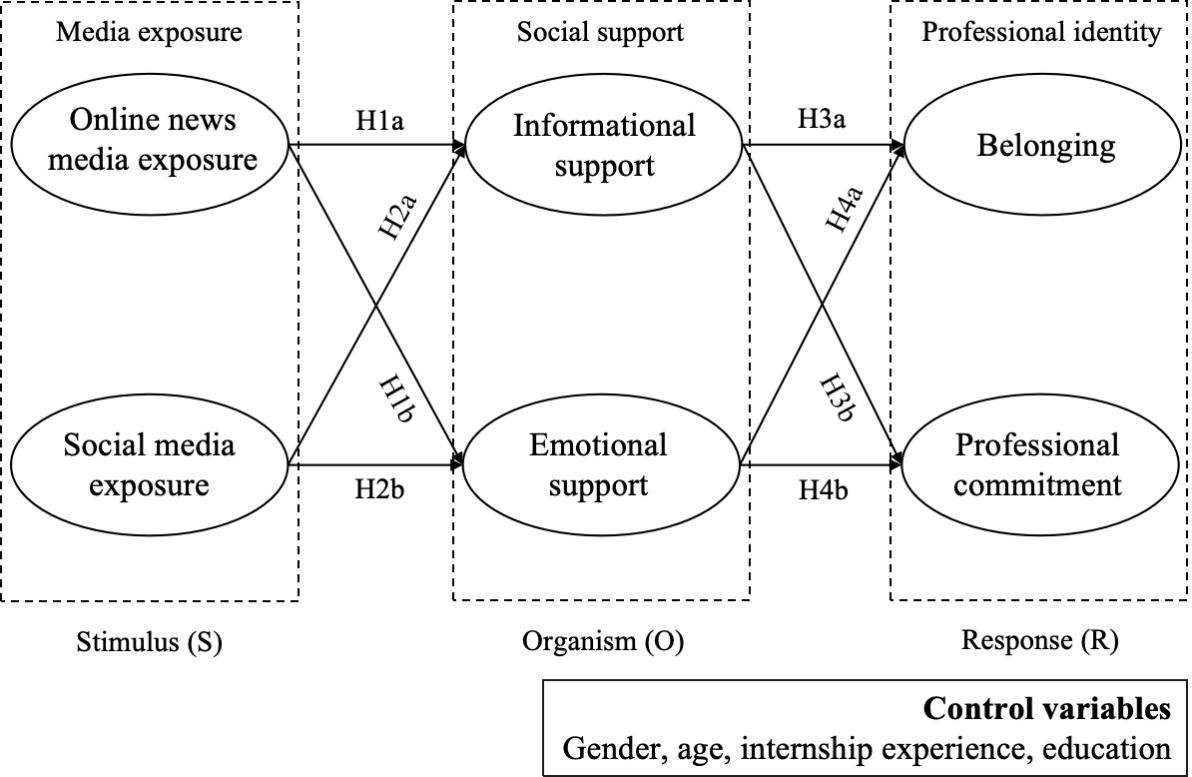
Research model.

This study also included gender, age, education, and early internship experience as control variables, as they may affect medical students’ professional identity [8,12]. The research model is depicted in Figure 1.

## Methods

### Survey Design

To empirically test the proposed research model, a cross-sectional survey was conducted to collect data. The survey design adhered to the CHERRIES (Checklist for Reporting Results of Internet E-Surveys) checklist [70] (see Multimedia Appendix 1 for the completed CHERRIES). A convenience sampling method was used, inviting medical students who were readily available to participate. Participants were recruited with the assistance of faculty members at a medical college. Specifically, the study team shared information about the survey in several medical courses and invited students to participate voluntarily. They were informed that their anonymity would be ensured to encourage honest responses and reduce social desirability bias [71]. The survey was conducted in May 2022.

Overall, the main body of the questionnaire consisted of 4 sections: (1) media exposure; (2) social support; (3) professional identity; and (4) demographic characteristics. Additionally, an introductory page was included at the beginning of the questionnaire to inform participants about the research purpose, target population, length of the survey, and policies regarding data security and storage. Furthermore, all participants were required to provide informed consent.

### Ethical Considerations

The questionnaire and methodology for this study were approved by the Ethics Committee of Tongji Medical College of Huazhong University of Science and Technology (approval number 2022S009). Informed consent was obtained from all participants. Measures were implemented to protect the privacy of all participants and ensure the confidentiality of the data. The questionnaire was anonymous, and the data file was stored on a secure drive accessible only to research team members who signed a confidentiality agreement. Each participant received a compensation of 10 RMB (approximately US $1.5) for their valid response.

### Measurement Development

This study used several strategies to develop the measurement instruments. First, to ensure the content validity of the instrument, measures for the variables were primarily adapted from well-validated scales and modified to fit the Chinese context and the characteristics of medical students. The back-translation method was used to adapt the original English scale to Chinese and ensure consistency between the 2 language versions. Specifically, the questions were first framed in English, then translated into Chinese, and finally back-translated into English to confirm consistency.

Second, we conducted several rounds of pretests to refine the instruments. Three professors and a group of graduate students from a medical school were invited to participate in these pretests. They completed the questionnaires and took part in follow-up interviews to evaluate whether the instruments effectively measured the constructs under investigation. Participants were also asked to provide feedback on the wording of the questions, clarity of expression, and question sequence. Based on their feedback, we enhanced the face validity and content validity of the instruments.

Third, we conducted a pilot test to assess the reliability and validity of the instruments. The results of the data analysis (shown in Multimedia Appendix 2; also see [72-75]) indicated that all measurements demonstrated ideal reliability and validity. Additionally, the measurement model analysis in the formal test further confirmed the reliability and validity of the instruments. The measures for the constructs are provided as follows.

### Measures

#### Social Media Exposure and Online News Media Exposure

The measures for media exposure were adapted from Gao et al [76]. Five items were used to assess medical students’ media exposure on a 7-point Likert scale (1=never to 7=very often). Participants were asked to evaluate the frequency of their exposure to information about medical workers and the medical profession (eg, health information from medical professionals, news stories or comments about medical workers, or information regarding medical education) on online news media and social media during the COVID-19 pandemic.

Considering the Chinese media environment and the media usage habits of medical students, this study included common news media (eg, Xinhuanet and Tencent News) as well as medical news media (eg, Medsci and PubMed) as components of online news media. Social media included Sina Weibo (Weibo Corporation), WeChat (Tencent Holdings Limited), and QQ (Shenzhen Tencent Computer System Co., Ltd.), as these 3 platforms are representative applications among Chinese youth [77].

#### Informational Support and Emotional Support

Informational support and emotional support were measured using items adapted from Nick et al [59]. Three items were used to assess each type of support through a 7-point Likert scale (1=never to 7=very often). To measure informational support, participants were asked to evaluate the frequency with which they encountered the following situations online in the past year: (1) people provided me with helpful medical information online; (2) I received help from others online when I had difficulties with my medical learning; and (3) people helped me understand my profession better while I was online. To measure emotional support, participants were asked to evaluate the frequency with which they encountered the following situations on the internet in the past year: (1) people showed that they cared about medical workers online; (2) people supported the work of medical workers online; and (3) individuals on the internet liked the things that medical workers said or did.

#### Belonging and Professional Commitment

The existing literature often uses self-reported measures to evaluate professional identity. For instance, Heidari et al [78] used a cross-sectional survey to examine the effects of online social capital and social networking on the formation of students’ professional identities. Similarly, Zhang et al [79] conducted an online cross-sectional survey to explore the relationship between psychological resilience and the sense of professional identity. Researchers typically regard professional identity as a psychological construct that emphasizes individuals’ professional self-concept [6]. As professional identity is a complex structure [31] that encompasses individuals’ feelings and behaviors toward their profession [32], this study uses belonging and professional commitment as indicators of professional identity and measures medical students’ feelings and behavioral intentions toward the profession.

Belonging was measured using items adapted from Lin et al [80]. Participants were asked to indicate the extent to which they agree with the following statements, reflecting their social affiliation and emotional connection with the medical group: (1) I belong to the medical group; (2) I feel socially connected to medical workers; and (3) I will be part of the medical group in the future. All items were measured on a 7-point Likert scale (1=strongly disagree to 7=strongly agree).

As mentioned above, professional commitment consists of 3 dimensions. Despite their conceptual differences, these dimensions are simultaneously experienced by individuals [38], reflecting their attitudes and behavioral intentions. Therefore, it is appropriate to measure these 3 dimensions as components of professional commitment. The measurement instruments were adapted from Meyer et al [36] and modified to align with the medical profession. Participants were asked to rate their level of agreement with the following statements: (1) I feel proud to be a medical worker and have no regrets about pursuing the medical profession; (2) I do not regret entering the medical profession; (3) It would be costly for me to change my profession now; (4) There are no pressures preventing me from changing my profession; and (5) I feel a responsibility to continue in the medical profession. Notably, the first 2 items represent affective commitment, while the third and fourth items reflect normative commitment. The last item is designed to capture participants’ continuance commitment. All items were measured on a 7-point Likert scale (1=strongly disagree to 7=strongly agree).

### Data Collection and Samples

Medical students from 4 majors (clinical medicine, basic medicine, nursing, and public health) at a medical college in China were recruited to complete a set of paper questionnaires. We selected these 4 majors due to their critical roles in the frontlines of the COVID-19 pandemic. Specifically, doctors, nurses, and epidemiologists from clinical medicine, nursing, and public health actively participated in the prevention and control of the pandemic. Students majoring in basic medicine were also included, as they play a vital role in combating the new coronavirus. By choosing a diverse range of majors, we aimed to enhance the generalizability of this study.

In the formal data collection process, a screening question was included to allow only medical students with online media usage experience to participate in the survey. Notably, participants from the pilot test were not permitted to complete the questionnaire. The survey included questions regarding demographic information, professional identity, and factors related to media exposure and social support. Additionally, we incorporated attention-trap questions to ensure the quality of the responses. A total of 1200 questionnaires were collected. After removing 113 invalid responses (eg, those that failed the attention test, contained missing values, or had excessively duplicated answers), we finalized a sample of 1087 valid responses, with students from the 4 majors approximately evenly distributed.

### Data Analysis

This study used structural equation modeling to test the research framework. We selected SmartPLS 4.0 (SmartPLS GmbH), based on partial least squares, as it allows for simultaneous testing of both the measurement model and the structural model [81]. In the current model, online news media exposure and social media exposure were incorporated as independent variables; informational support and emotional support served as mediators; and professional identity (ie, belonging and professional commitment) variables were included as outcomes. Following the approach suggested by Anderson and Gerbing [82], we first analyzed the measurement model to assess reliability and validity, and subsequently conducted structural model analysis to evaluate our research model. Furthermore, a mediation analysis was conducted to uncover the underlying mechanisms of media exposure’s influence. To account for potential confounding effects, gender, age, education, and internship experience were included as control variables in the structural equation model.

## Results

### Demographic Information

As mentioned above, a final sample of 1087 valid responses was obtained. The demographic information of this sample is summarized in Table 1. More than half of the students were female and pursuing an undergraduate degree. Additionally, most of the students were in their 20s or younger and reported having internship experiences during their studies.

**Table 1:** Demographic information (N=1087).

Characteristic		Values, n (%)
**Gender**		
	Male	461 (42.41)
	Female	626 (57.59)
**Age (years)**		
	<22	701 (64.49)
	23-25	278 (25.57)
	26-29	101 (9.29)
	30-40	7 (0.64)
**Internship (years)**		
	0	252 (23.18)
	0.5	327 (30.08)
	0.6-1.5	306 (28.15)
	1.6-3	179 (16.47)
	>3	23 (2.12)
**Education**		
	Undergraduate	756 (69.55)
	Master’s	221 (20.33)
	Doctor	110 (10.12)

### Measurement Model Test

This study assesses the convergent and discriminant validity to evaluate the measurement model. The descriptive statistics of the variables in our research model and the results are presented in Table 2. Notably, Cronbach α for all constructs exceeded 0.7. Additionally, the composite reliability values for all constructs were greater than 0.8, and all average variance extracted (AVE) values were above 0.5, meeting the recommended threshold values of 0.7 and 0.5, respectively [72,73]. In addition, all item loadings, except for the one related to professional commitment, exceeded 0.7. As this factor loading was close to 0.7, we retained it to consider content validity. Thus, the model demonstrates acceptable reliability and convergent validity. We further assessed discriminant validity by comparing the interconstruct correlation coefficients with the square roots of AVE [72]. The results in Table 3 indicate that the square roots of AVE exceed all interconstruct correlation coefficients, confirming that discriminant validity is not an issue.

This study also examined multicollinearity. As a rule, multicollinearity is considered high if the variance inflation factor of a variable exceeds 10 [72]. In this study, the variance inflation factor values for all variables were below 3, indicating the absence of significant multicollinearity. Additionally, because all data were collected from a cross-sectional survey, common method bias (CMB) was assessed. First, we conducted the Harman single-factor test using SPSS Statistics 26.0 (IBM Corp.) and found that the most important factor explained only 23.33% of the total variance, which is lower than the reference value of 50% [74]. Second, CMB may lead to high correlations between constructs. Table 3 shows that the highest correlation between constructs was 0.675, which is below the recommended threshold of 0.90 [75]. Therefore, CMB was not a significant threat in this study.

**Table 2:** Reliability and convergent validity analysis.

Item		VIF^a^	Item loading	Mean (SD)	AVE^b^	CR^c^	Cronbach α
**Online news** **media exposure**					0.768	0.868	0.713
	Online news media exposure 1	1.442	0.935	3.74 (1.82)			
	Online news media exposure 2	1.442	0.814	3.34 (1.78)			
**Social media exposure**					0.647	0.845	0.726
	Social media exposure 1	1.323	0.733	4.49 (2.04)			
	Social media exposure 2	1.878	0.909	4.92 (1.68)			
	Social media exposure 3	1.571	0.759	4.38 (1.81)			
**Informational support**					0.755	0.902	0.838
	Informational support 1	2.107	0.892	5.12 (1.30)			
	Informational support 2	2.017	0.857	5.02 (1.34)			
	Informational support 3	1.837	0.858	5.18 (1.29)			
**Emotional support**					0.752	0.900	0.834
	Emotional support 1	2.189	0.874	5.53 (1.31)			
	Emotional support 2	2.785	0.918	5.58 (1.25)			
	Emotional support 3	1.700	0.805	5.29 (1.40)			
**Belonging**					0.804	0.925	0.878
	Belonging 1	2.343	0.888	5.05 (1.59)			
	Belonging 2	2.618	0.919	5.52 (1.39)			
	Belonging 3	2.333	0.883	5.29 (1.55)			
**Professional commitment**					0.581	0.873	0.819
	Professional commitment 1	1.999	0.835	5.16 (1.49)			
	Professional commitment 2	2.083	0.789	4.69 (1.60)			
	Professional commitment 3	1.484	0.678	5.40 (1.56)			
	Professional commitment 4	1.668	0.757	5.60 (1.47)			
	Professional commitment 5	1.747	0.743	4.73 (1.57)			

^a^VIF: variance inflation factor.

^b^AVE: average variance extracted.

^c^CR: composite reliability.

**Table 3:** Discriminant validity analysis.

Analyzed variables	Online news media exposure	Social media exposure	Informational support	Emotional support	Belonging	Professional commitment
Online news media exposure	*0* *.876* ^a^	—^b^	—	—	—	—
Social media exposure	0.260	*0.804* ^a^	—	—	—	—
Informational support	0.195	0.164	*0.869* ^a^	—	—	—
Emotional support	0.145	0.256	0.593	*0.867* ^a^	—	—
Belonging	0.160	0.188	0.437	0.473	*0.897* ^a^	—
Professional commitment	0.165	0.189	0.494	0.552	0.675	*0.762* ^a^

^a^The square roots of average variances extracted are in italics.

^b^Not applicable.

### Structural Model Test

**Figure 2:**
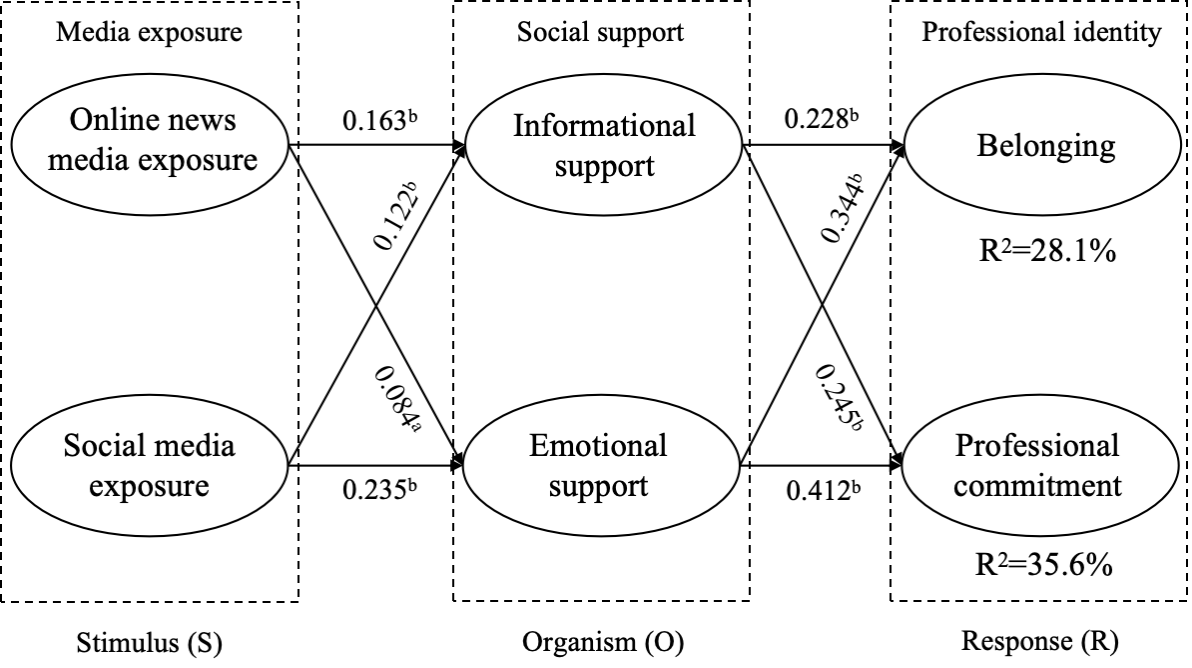
Data analysis results. ^a^*P*<.01; ^b^*P*<.001.

The results of the structural model test are shown in Figure 2. Consistent with our expectations, online news media exposure was positively associated with informational support (β=.163; *P*<.001) and emotional support (β=.084; *P*=.007). Additionally, social media exposure was positively associated with informational support (β=.122; *P*<.001) and emotional support (β=.235; *P*<.001). Therefore, H1a, H1b, H2a, and H2b are all supported.

Thereafter, this study analyzed the impact of social support on medical students’ professional identity. The results showed that informational support (β=.228; *P*<.001) and emotional support (β=.344; *P*<.001) were positively associated with belonging. Meanwhile, we found that informational support (β=.245; *P*<.001) and emotional support (β=.412; *P*<.001) were positively associated with medical students’ professional commitment. Overall, the model explained 28.1% of the variance in belonging and 35.6% of the variance in professional commitment. Therefore, H3a, H3b, H4a, and H4b are all supported.

This study also tested the impact of demographic variables on medical students’ professional identity. The results showed that the impacts of gender on belonging (β=.064; *P*=.22) and professional commitment (β=–.008; *P*=.87) were insignificant. Meanwhile, the impacts of age on belonging (β=–.074; *P*=.08) and professional commitment (β=–.053; *P*=.17) were also insignificant. Furthermore, the impacts of education on belonging (β=–.061; *P*=.15) and professional commitment (β=–.022; *P*=.54) were likewise insignificant. Notably, internship experience had positive effects on both belonging (β=.114; *P*<.001) and professional commitment (β=.091; *P*<.001).

### Mediation Analysis

The mediation effects of social support were further assessed following Hair et al [83] and Zhao et al [84]. Table 4 shows the results of our mediation analysis. The results indicated that specific indirect effects for both independent variables were significant, confirming the mediation effect of social support. Additionally, online news media exposure had significant direct effects on both belonging (β=.076; *P*=.01) and professional commitment (β=.059; *P*=.03). Therefore, informational support and emotional support partially mediated the relationship between online news media exposure and medical students’ professional identity [81]. Thus, H5a, H5b, H6a, and H6b were supported. In addition, as the direct effects of social media on belonging (β=.060; *P*=.06) and professional commitment (β=.045; *P*=.13) were both insignificant, social support fully mediated the relationship between social media exposure and medical students’ professional identity [81]. As such, H7a, H7b, H8a, and H8b were all supported.

**Table 4:** Results of the mediation analysis.

Independent variable and relationship		Specific indirect effect (*P* value)	Direct effect (*P* value)	Mediation effect
**Online news** **media exposure**				
	Online news media exposure→Informational support→Belonging	0.035 (<.001)	0.076 (.01)	Partial mediation
	Online news media exposure→Emotional support→Belonging	0.029 (.011)	0.076 (.01)	Partial mediation
	Online news media exposure→Informational support→Professional commitment	0.039 (<.001)	0.059 (.03)	Partial mediation
	Online news media exposure→Emotional support→Professional commitment	0.035 (.007)	0.059 (.03)	Partial mediation
**Social media exposure**				
	Social media exposure→Informational support→Belonging	0.026 (.002)	0.060 (.06)	Full mediation
	Social media exposure→Emotional support→Belonging	0.076 (<.001)	0.060 (.06)	Full mediation
	Social media exposure→Informational support→Professional commitment	0.029 (<.001)	0.045 (.13)	Full mediation
	Social media exposure→Emotional support→Professional commitment	0.092 (<.001)	0.045 (.13)	Full mediation

## Discussion

### Principal Findings

In light of the far-reaching crisis brought by the pandemic, this study aims to uncover how media factors can help mitigate social risks within the field of medical sociology. Utilizing the SOR framework, we propose a research model to examine the impacts of media exposure on medical students’ professional identity. Our findings reveal several intriguing insights.

First and foremost, our findings indicate that both online news media exposure and social media exposure are positively related to perceived emotional and informational support. This suggests that medical students can derive both types of support from these media sources. Notably, the path coefficient between online news media and informational support was relatively higher than that for emotional support, whereas the opposite was true for social media exposure. The results can be explained by the inherent characteristics of the 2 types of media. Online news media are generally perceived as trustworthy due to their strict gatekeeping mechanisms [2], making them vital and reliable sources for informational support. By contrast, social media serves as an important platform for facilitating online interactions [46], thereby proving effective in providing emotional support.

Second, emotional support and informational support are positively associated with belonging and professional commitment, indicating that social support is a significant predictor of medical students’ professional identity. Notably, the path coefficients for emotional support on both belonging and professional commitment are higher than those for informational support. The results suggest that emotional support may have a greater impact on fostering medical students’ professional identity than informational support. This finding aligns with Wang et al [61], who emphasized the importance of emotional support in enhancing interpersonal relationships within online health support groups. Additionally, this can be explained by the nature of professional identity itself; both belonging and professional commitment relate to individuals’ feelings toward their professional group and the profession [37,38], making them more likely to be driven by emotional factors.

Third, the impact of media exposure on professional identity was mediated by social support. Specifically, social support partially mediated the effect of online news media exposure and fully mediated the effect of social media exposure. These results indicate that social media primarily relies on the provision of social support to influence professional identity, whereas online news media exert their effects on professional identity both directly and indirectly through social support. One possible explanation for this finding lies in the characteristics of different media. During the pandemic, individuals primarily relied on social media to transmit and obtain social support. By contrast, by reporting news about medical workers, online news media can directly assist medical students in developing their understanding of professional identity [19]. These findings further clarify that social media and online news media might play different roles in shaping medical students’ professional identity.

Fourth, the analysis of control variables shows that internship experience is positively associated with medical students’ belonging and professional commitment. For medical students, the medical profession places great emphasis on practicality, making internships an essential avenue for gaining knowledge about the field. On the one hand, internships help students develop their self-efficacy by providing hands-on experience and exposure to real-world medical environments [85]; on the other hand, practical experience can enhance medical students’ awareness of the profession [86]. Consequently, the training received during internships may better prepare students to become medical workers, which, in turn, inspires them to cultivate a sense of belonging and professional commitment to the field.

### Implications

The internet has emerged as the predominant form of media globally, with social media serving as a crucial platform during the COVID-19 pandemic [3]. Given the media environment’s significant influence on shaping the cognition and behaviors of the younger generation [87], as well as the importance of medical students’ professional identity for the stability of the medical community, this study explores the impact of media exposure on medical students’ professional identity and seeks to uncover the mechanisms underlying this relationship. By comparing with prior work, this study offers several theoretical implications. First, it enriches the existing literature on professional identity in medical education. Previous studies typically focused on individual and educational environmental factors in the development of professional identity [12-14], often neglecting the role of the media environment. By integrating media exposure research with a social psychology perspective, this study empirically validates a new set of relationships and illustrates how media exposure influences medical students’ perceived social support and professional identity. Unlike prior work, this study examines 2 types of media exposure simultaneously, developing a nuanced understanding of the effects of online news media and social media on medical education. The findings emphasize the significance of media in fostering medical students’ professional identity, offering a new perspective for understanding the development of professional identity.

Second, this study extends the application of the SOR framework to explore the psychological processes underlying the development of professional identity, complementing the literature that examines the direct impacts of social factors on professional identity. It demonstrates that media exposure influences professional identity through both informational support and emotional support. Previous research has tended to focus on either cognitive or affective aspects of the organism component in the SOR framework [39]. This study further differentiates these 2 aspects by discussing the distinct impacts of informational support and emotional support on professional identity. Additionally, by illustrating how social support can be generated from different types of media exposure, this study contributes to the literature on the development of social support within various media environments.

This study also has practical implications for media platforms and medical educators. Above all, the findings confirm the importance of the media environment in fostering professional identity. These results are particularly relevant in the context of the Chinese media landscape. Since the outbreak of the COVID-19 pandemic, a substantial amount of information sharing praise and rewards for medical workers has circulated on Chinese media platforms. Exposure to such information may help students recognize the public’s appreciation for their profession, thereby enhancing their perception of social support as well as their professional identity. Consequently, the proper use and management of media during public health crises can facilitate the development of medical students’ professional identity. Considering the differences between online news media and social media, management departments and medical educators can leverage online news media to post reliable medical information, providing informational support, while encouraging favorable social interactions through social media to offer emotional support.

Additionally, our findings emphasize the importance of social support in enhancing professional identity. Sufficient social support should be provided to improve students’ well-being during their learning experiences [88]. By alleviating mental health issues such as depression, stress, and anxiety, students’ professional identity can be further promoted [89]. Furthermore, it is beneficial to present positive images of medical workers in online media so that medical students can develop a positive self-concept about the medical profession [77]. When reporting on medical workers, journalists should emphasize the value of medical professionalism rather than simply portraying them as angels or heroes [90,91]. In addition to focusing on providing social support through online media, medical educators and management departments can implement other measures to support students in coping with the challenges they encounter and improving their well-being, thereby reinforcing their professional identity.

Moreover, the significant effects of internships on belonging and professional commitment offer valuable implications for medical educators. Specifically, medical educators should provide opportunities for medical students to engage in practical learning. However, given that many medical students often have limited experience interacting with patients, leveraging media resources to provide professional knowledge can serve as an important complement to their internships. The practical experience gained from internships and the knowledge acquired through online media can be combined to motivate medical students to feel a sense of belonging and commitment to their profession.

### Limitations and Future Research

There are several limitations that warrant future research. First, our study is designed based on the media environment in China. While the data illustrate the impacts of media exposure on the professional identity of Chinese medical students, future research could recruit respondents from other countries to enhance the generalizability of the findings. Second, this study focuses on online news media and social media without considering other media forms, such as short video platforms. Future research can consider the effects of other types of media. Third, this study collected self-reported data through a cross-sectional survey. While the relationships between variables can be tested, this approach limits the ability to validate causal relationships. Future studies could adopt longitudinal designs to collect data and examine the effects of media exposure. In addition, self-reported data may be less reliable in predicting actual behavior. Future research should consider using objective measures to assess professional identity. Fourth, while this study focuses on media exposure to uncover its impacts on the development of medical students’ professional identity, it is important to acknowledge that professional identity can be influenced by various factors. Future studies should explore additional factors in different contexts, particularly those that may hinder the development of professional identity.

### Conclusions

Whether faced with a plague or war, health workers have always been on the front lines of combating social risks. Their professional identity serves as both a foundation for their beliefs and a source of strength. As members of the young generation in the age of social media, medical students’ cognitions and behaviors are easily shaped by the media environment [87]. It is thus urgent to conduct in-depth research on the impacts of media exposure on medical students’ professional identity, especially as the global wave of medical staff resignations intensifies. This study extends the SOR framework to examine the effects of media exposure related to long COVID on medical students’ professional identity, aiming to provide insights into the critical issue of medical workforce shortages. Based on a survey of 1087 medical students, this study reveals that exposure to online news media and social media can enhance medical students’ sense of belonging and professional commitment through emotional and informational support. It clarifies the role of media affordances in medical education, underscores the importance of media management and social support in shaping professional identity, and expands the understanding of the media’s role in public health events. In the new media environment, these factors may become crucial components and governance tools in medical sociology, influencing health outcomes and serving as a viable approach to achieving the One Health goal in the long COVID era.

## Appendix

Multimedia Appendix 1 CHERRIES (Checklist for Reporting Results of Internet-Based e-Surveys) checklist.

Multimedia Appendix 2 Data analysis results of the pilot test.

## Abbreviations

AVE: average variance extracted
CHERRIES: Checklist for Reporting Results of Internet E-Surveys
CMB: common method bias
SOR: Stimulus-Organism-Response

## Data Availability

The data sets generated during or analyzed during this study are available from the corresponding author upon reasonable request.

## References

[1] Ren W, Zhu X, Hu Y (2021). Risk perception and preventive behaviors: a comparison of the multifaceted effects of social media and authoritative media during the outbreak of COVID-19. Chin J Journal Commun.

[2] Du Z, Luo X, Su L (2022). Collective action under social expectation: the cognitive construction of media exposure on COVID-19 vaccination intention. Chin J Journal Commun.

[3] World Health Organization (WHO) Managing the COVID-19 infodemic: promoting healthy behaviours and mitigating the harm from misinformation and disinformation. WHO.

[4] World Health Organization (WHO) Global strategy on human resources for health: workforce 2030: reporting at Seventy-fifth World Health Assembly. WHO.

[5] Passemard S, Faye A, Dubertret C, Peyre H, Vorms C, Boimare V, Auvin S, Flamant M, Ruszniewski P, Ricard J (2021). Covid-19 crisis impact on the next generation of physicians: a survey of 800 medical students. BMC Med Educ.

[6] Tempski P, Arantes-Costa FM, Kobayasi R, Siqueira MAM, Torsani MB, Amaro BQRC, Nascimento MEFM, Siqueira SL, Santos IS, Martins MA (2021). Medical students' perceptions and motivations during the COVID-19 pandemic. PLoS One.

[7] Yan J, Wu M, Liao Y, Huang Y (2024). Modelling the factors that affect medical students’ occupational identity in long COVID: an integrated perspective of motivation, opportunity and ability. Humanit Soc Sci Commun.

[8] Johnson M, Cowin LS, Wilson I, Young H (2012). Professional identity and nursing: contemporary theoretical developments and future research challenges. Int Nurs Rev.

[9] Erikson H (1968). Identity, Youth, and Crisis.

[10] Shu M, Huang S, Chen J (2019). Impact of medical practice environmental assessment on professional identity of clinical medical students. Med Soc.

[11] Sun L, Liang W, Mei Y, Lin L, Yu Y, Xia O (2015). Investigation on the professional identity of master degree postgraduate of clinical medicine. Chin J Med Educ.

[12] Helmich E, Derksen E, Prevoo M, Laan R, Bolhuis S, Koopmans R (2010). Medical students' professional identity development in an early nursing attachment. Med Educ.

[13] Cavenagh P, Dewberry C, Jones P (2000). Becoming professional: when and how does it start? A comparative study of first-year medical and law students in the UK. Med Educ.

[14] Duran S, Celik I, Ertugrul B, Ok S, Albayrak S (2021). Factors affecting nurses' professional commitment during the COVID-19 pandemic: a cross-sectional study. J Nurs Manag.

[15] Ruan B, Yilmaz Y, Lu D, Lee M, Chan TM (2020). Defining the digital self: a qualitative study to explore the digital component of professional identity in the health professions. J Med Internet Res.

[16] De Coninck D, d'Haenens L, Matthijs K (2020). Forgotten key players in public health: news media as agents of information and persuasion during the COVID-19 pandemic. Public Health.

[17] Perez‐Lugo M (2004). Media uses in disaster situations: a new focus on the impact phase. Sociological Inquiry.

[18] Krawczyk K, Chelkowski T, Laydon DJ, Mishra S, Xifara D, Gibert B, Flaxman S, Mellan T, Schwämmle Veit, Röttger Richard, Hadsund JT, Bhatt S (2021). Quantifying online news media coverage of the COVID-19 pandemic: text mining study and resource. J Med Internet Res.

[19] Bagnasco Annamaria, Catania Gianluca, Gallagher Ann, Morley Georgina (2020). Media representations of nurses in the pandemic: just doing our job?. Nurs Ethics.

[20] Lee J, Kim K, Park G, Cha N (2021). The role of online news and social media in preventive action in times of infodemic from a social capital perspective: the case of the COVID-19 pandemic in South Korea. Telemat Inform.

[21] Bambina A (2007). Online Social Support: The Interplay of Social Networks and Computer-Mediated Communication.

[22] Porter CE, Donthu N (2008). Cultivating trust and harvesting value in virtual communities. Management Science.

[23] Gazzola N, De Stefano J, Audet C, Theriault A (2011). Professional identity among counselling psychology doctoral students: a qualitative investigation. Counselling Psychology Quarterly.

[24] Mehrabian A, Russell JA (1974). An Approach to Environmental Psychology.

[25] Browne C, Wall P, Batt S, Bennett R (2018). Understanding perceptions of nursing professional identity in students entering an Australian undergraduate nursing degree. Nurse Educ Pract.

[26] Loi R, Hang-yue N, Foley S (2004). The effect of professional identification on job attitudes: a study of lawyers in Hong Kong. Organ Anal.

[27] Zhang W, Meng H, Yang S, Liu D (2018). The influence of professional identity, job satisfaction, and work engagement on turnover intention among township health inspectors in China. Int J Environ Res Public Health.

[28] de Lasson L, Just E, Stegeager N, Malling B (2016). Professional identity formation in the transition from medical school to working life: a qualitative study of group-coaching courses for junior doctors. BMC Med Educ.

[29] Beijaard D, Meijer PC, Verloop N (2004). Reconsidering research on teachers’ professional identity. Teaching and Teacher Education.

[30] Brooks NG, Riemenschneider CK, Hardgrave BC, O'Leary-Kelly AM (2017). IT professional identity: needs, perceptions, and belonging. European Journal of Information Systems.

[31] Meijers F (1998). The development of a career identity. Int J Adv Couns Spe.

[32] Ashforth BE, Schinoff BS (2016). Identity under construction: how individuals come to define themselves in organizations. Annu Rev Organ Psychol Organ Behav.

[33] Patel SE, Chrisman M, Russell CL, Lasiter S, Bennett K, Pahls M (2022). Cross-sectional study of the relationship between experiences of incivility from staff nurses and undergraduate nursing students' sense of belonging to the nursing profession. Nurse Educ Pract.

[34] Aranya N, Ferris K (1984). A reexamination of accountants' organizational-professional conflict. Account Rev Jan.

[35] Lachman R, Aranya N (1986). Job attitudes and turnover intentions among professionals in different work settings. Organization Studies.

[36] Meyer JP, Allen NJ, Smith CA (1993). Commitment to organizations and occupations: extension and test of a three-component conceptualization. Journal of Applied Psychology.

[37] Hagerty BM, Lynch-Sauer J, Patusky KL, Bouwsema M, Collier P (1992). Sense of belonging: a vital mental health concept. Archives of Psychiatric Nursing.

[38] Meyer JP, Herscovitch L (2001). Commitment in the workplace: toward a general model. Human Resource Management Review.

[39] Eroglu SA, Machleit KA, Davis LM (2001). Atmospheric qualities of online retailing. Journal of Business Research.

[40] Luo P, Wang C, Guo F, Luo L (2021). Factors affecting individual online rumor sharing behavior in the COVID-19 pandemic. Comput Human Behav.

[41] Hewei T, Youngsook L (2022). Factors affecting continuous purchase intention of fashion products on social E-commerce: SOR model and the mediating effect. Entertainment Computing.

[42] Fu S, Li H, Liu Y, Pirkkalainen H, Salo M (2020). Social media overload, exhaustion, and use discontinuance: examining the effects of information overload, system feature overload, and social overload. Information Processing & Management.

[43] Zhang H, Lu Y, Gupta S, Zhao L (2014). What motivates customers to participate in social commerce? The impact of technological environments and virtual customer experiences. Information & Management.

[44] Pandita S, Mishra HG, Chib S (2021). Psychological impact of covid-19 crises on students through the lens of Stimulus-Organism-Response (SOR) model. Child Youth Serv Rev.

[45] Plantin L, Daneback K (2009). Parenthood, information and support on the internet. A literature review of research on parents and professionals online. BMC Fam Pract.

[46] Selkie E, Adkins V, Masters E, Bajpai A, Shumer D (2020). Transgender adolescents' uses of social media for social support. J Adolesc Health.

[47] Drew D, Weaver D (1990). Media attention, media exposure, and media effects. Journalism Quarterly.

[48] Gil de Zúñiga H, Molyneux L, Zheng P (2014). Social media, political expression, and political participation: panel analysis of lagged and concurrent relationships. J Commun.

[49] Brown J, L'Engle K, Pardun C, Guo G, Kenneavy K, Jackson C (2006). Sexy media matter: exposure to sexual content in music, movies, television, and magazines predicts black and white adolescents' sexual behavior. Pediatrics.

[50] Anderson P, de Bruijn A, Angus K, Gordon R, Hastings G (2009). Impact of alcohol advertising and media exposure on adolescent alcohol use: a systematic review of longitudinal studies. Alcohol Alcohol.

[51] Chao M, Xue D, Liu T, Yang H, Hall BJ (2020). Media use and acute psychological outcomes during COVID-19 outbreak in China. J Anxiety Disord.

[52] Kim A, Dennis AR (2019). Says who? The effects of presentation format and source rating on fake news in social media. MISQ.

[53] Rosen AO, Holmes AL, Balluerka N, Hidalgo MD, Gorostiaga A, Gómez-Benito Juana, Huedo-Medina TB (2022). Is social media a new type of social support? Social media use in Spain during the COVID-19 pandemic: a mixed methods study. Int J Environ Res Public Health.

[54] Langford CPH, Bowsher J, Maloney JP, Lillis PP (1997). Social support: a conceptual analysis. J Adv Nurs.

[55] Buschmann M, Hollinger L (1994). Influence of social support and control on depression in the elderly. Clinical Gerontologist.

[56] Oppong Asante K (2012). Social support and the psychological wellbeing of people living with HIV/AIDS in Ghana. Afr J Psychiatry (Johannesbg).

[57] Castellá Sarriera J, Bedin L, Abs D, Casas F, Calza T (2015). Relación entre soporte social, la satisfacción de vida y bienestar subjetivo en adolescentes brasileros. Univ Psychol.

[58] Lu W, Hampton KN (2016). Beyond the power of networks: differentiating network structure from social media affordances for perceived social support. New Media & Society.

[59] Nick EA, Cole DA, Cho S, Smith DK, Carter TG, Zelkowitz RL (2018). The online social support scale: measure development and validation. Psychol Assess.

[60] Ruiller C, Van Der Heijden BI (2016). Socio-emotional support in French hospitals: effects on French nurses' and nurse aides' affective commitment. Appl Nurs Res.

[61] Wang Y, Kraut R, Levine J (2012). To stay or leave? The relationship of emotionalinformational support to commitment in online health support groups. https://dl.acm.org/doi/abs/10.1145/2145204.2145329.

[62] Cronenwett LR (1985). Parental network structure and perceived support after birth of first child. Nursing Research.

[63] Sutherland S, Jalali A (2017). Social media as an open-learning resource in medical education: current perspectives. AMEP.

[64] Oh S, Lee SY, Han C (2021). The effects of social media use on preventive behaviors during infectious disease outbreaks: the mediating role of self-relevant emotions and public risk perception. Health Commun.

[65] Bellanti F, Lo Buglio A, Capuano E, Dobrakowski M, Kasperczyk A, Kasperczyk S, Ventriglio A, Vendemiale G (2021). Factors related to nurses’ burnout during the first wave of coronavirus disease-19 in a university hospital in Italy. IJERPH.

[66] Chang H, Shyu YL, Wong M, Chu T, Lo Y, Teng C (2017). How does burnout impact the three components of nursing professional commitment?. Scand J Caring Sci.

[67] Ahmad Z, Anantharaman RN, Ismail H (2012). Students' motivation, perceived environment and professional commitment: an application of Astin's college impact model. Accounting Education.

[68] Kjærgaard A, Morsing M, Ravasi D (2011). Mediating identity: a study of media influence on organizational identity construction in a celebrity firm. J Manage Stud.

[69] Geusens F, Beullens K (2023). I see, therefore I am: exposure to alcohol references on social media, but not on traditional media, is related to alcohol consumption via drinking and non-drinking identity. Health Commun.

[70] Eysenbach Gunther (2004). Improving the quality of Web surveys: the Checklist for Reporting Results of Internet E-Surveys (CHERRIES). J Med Internet Res.

[71] Burns AJ, Roberts TL, Posey C, Lowry PB, Fuller B (2023). Going beyond deterrence: a middle-range theory of motives and controls for insider computer abuse. Information Systems Research.

[72] Fornell C, Larcker DF (1981). Structural equation models with unobservable variables and measurement error: algebra and statistics. Journal of Marketing Research.

[73] Hair JF, Black WC, Babin BJ, Anderson RE (2010). Multivariate Data Analysis: A Global Perspective (7th Edition).

[74] Harman HH (1976). Modern Factor Analysis.

[75] Bagozzi RP, Yi Y, Phillips LW (1991). Assessing construct validity in organizational research. Administrative Science Quarterly.

[76] Gao J, Zheng P, Jia Y, Chen H, Mao Y, Chen S, Wang Y, Fu H, Dai J (2020). Mental health problems and social media exposure during COVID-19 outbreak. PLoS One.

[77] Administration Center of China E-Government Network, Online Communication Research Institute of Nanjing University A research on public awareness and information dissemination of novel coronavirus pneumonia was officially released. Administration Center of China E-Government Network.

[78] Heidari E, Salimi G, Mehrvarz M (2020). The influence of online social networks and online social capital on constructing a new graduate students’ professional identity. Interactive Learning Environments.

[79] Zhang Z, Fu W, Tian C, Zhang F, Zhao B, Mao J, Saligan LN (2021). Professional identity of Chinese nursing students during the COVID-19 pandemic outbreak: a nation-wide cross-sectional study. Nurse Education in Practice.

[80] Lin H, Fan W, Chau PY (2014). Determinants of users’ continuance of social networking sites: a self-regulation perspective. Information & Management.

[81] Urbach N, Ahlemann F (2010). Structural equation modeling in information systems research using partial least squares. J Inform Technology Theory Appl.

[82] Anderson JC, Gerbing DW (1988). Structural equation modeling in practice: a review and recommended two-step approach. Psychological Bulletin.

[83] Hair JF, Hult GTM, Ringle CM, Sarstedt MA (2021). A Primer on Partial Least Squares Structural Equation Modeling (PLS-SEM) (3rd Edition).

[84] Zhao X, Lynch JG, Chen Q (2010). Reconsidering Baron and Kenny: myths and truths about mediation analysis. J Consum Res.

[85] Lang JC, Lee CH (2005). Identity accumulation, others' acceptance, job-search self-efficacy, and stress. J Organiz Behav.

[86] Filstad C, Traavik LE, Gorli M (2019). Belonging at work: the experiences, representations and meanings of belonging. JWL.

[87] Purba AK, Henery PM, Thomson RM, Pearce A, Henderson M, Katikireddi SV (2021). Social media use and adolescent engagement in health risk behaviours: a systematic review and meta-analysis. The Lancet.

[88] Guo K, Zhang X, Bai S, Minhat HS, Nazan AINM, Feng J, Li X, Luo G, Zhang X, Feng J, Li Y, Si M, Qiao Y, Ouyang J, Saliluddin S (2021). Assessing social support impact on depression, anxiety, and stress among undergraduate students in Shaanxi province during the COVID-19 pandemic of China. PLoS One.

[89] Hao C, Zhu L, Zhang S, Rong S, Zhang Y, Ye J, Yang F (2020). Serial multiple mediation of professional identity, and psychological capital in the relationship between work-related stress and work-related well-being of ICU nurses in China: a cross-sectional questionnaire survey. Front Psychol.

[90] Halberg N, Jensen PS, Larsen TS (2021). We are not heroes-The flipside of the hero narrative amidst the COVID19-pandemic: a Danish hospital ethnography. J Adv Nurs.

[91] Stokes-Parish Jessica, Elliott R, Rolls K, Massey D (2020). Angels and heroes: the unintended consequence of the hero narrative. J Nurs Scholarsh.

