# Supplementary material for: Impact of Concurrent Media Exposure on Professional Identity: Cross-Sectional Study of 1087 Medical Students During Long COVID
Source: J Med Internet Res. 2024 Oct 17;26:e50057. doi: 10.2196/50057 (PMC11528167; doi:10.2196/50057)
Supplement: Multimedia Appendix 1 [file jmir_v26i1e50057_app1.docx]

**Multimedia Appendix 1: CHERRIES (Checklist for Reporting Results of Internet-Based e-Surveys) checklist.**

| ***Item Category*** | ***Checklist Item*** | ***Explanation*** |
| --- | --- | --- |
| **Design** |  |  |
|  | Describe survey design | See ‘Survey Design’ and ‘Data Collection and Samples’ |
| **IRB (Institutional Review Board) approval and informed consent process** | IRB approval | See ‘Ethical Considerations’  The questionnaire and methodology for this study was approved by the Ethics Committee of Tongji Medical College of Huazhong University of Science and Technology (No. 2022S009). |
|  | Informed consent | See ‘Ethical Considerations’  The informed consent was obtained from all participants. |
|  | Data protection | See ‘Ethical Considerations’  Measures were taken to protect privacy of all the participants and guarantee the confidentiality of the data. The questionnaire was anonymous, and the data file was stored on the secured drive of the research team. Only research members of the team, who signed a confidentiality agreement, were able to view and analyze the data file. |
| **Development and pre-testing** | Development and testing | See ‘Measurement Development’  This study took several measures to develop the measurement instruments.  We also conducted a pilot test to assess the reliability and validity of the instruments. |
| **Recruitment process and description of the sample having access to the questionnaire** | Open survey versus closed survey | See ‘Data Collection and Samples’  Open survey. Participants can answer the questionnaire voluntarily by getting the questionnaire from the teachers. |
|  | Contact mode | See ‘Data Collection and Samples’  Participants were contacted with the assistance of teachers in a medical college in China. |
|  | Advertising the survey | No advertisement was promoted. |
| **Survey administration** | Web/E-mail | N/A |
|  | Context | N/A |
|  | Mandatory/voluntary | Voluntary |
|  | Incentives | 10 RMB (approximately 1.5 USD) |
|  | Time/Date | Data was collected in May 2022. |
|  | Randomization of items or questionnaires | Question order was not randomized. |
|  | Adaptive questioning | N/A |
|  | Number of Items | 23 items |
|  | Number of screens (pages) | N/A |
|  | Completeness check | There was no completeness check before submission. Therefore, some answers are missing. We removed questionnaire with missing values. |
|  | Review step | Participants were able to review and change their answers before they submit the questionnaire. |
| **Response rates** |  |  |
|  | Unique site visitor | N/A |
|  | View rate (Ratio of unique survey visitors/unique site visitors) | N/A |
|  | Participation rate (Ratio of unique visitors who agreed to participate/unique first survey page visitors) | N/A |
|  | Completion rate (Ratio of users who finished the survey/users who agreed to participate) | In this study, participants filled in the questionnaire on behalf of their consent to participate.  Despite several questionnaires have been found to have missing values for some single items, we didn’t regard these questionnaires uncompleted because very most of the other items were well answered. As such, completion rate could not be calculated here. Indeed, we attributed these missing values for absence of patience and attention and excluded them in statistical analysis. |
| **Preventing multiple entries from the same individual**^a^ | Cookies used | N/A |
|  | IP check | N/A |
|  | Log file analysis | N/A |
|  | Registration | N/A |
| **Analysis** | Handling of incomplete questionnaires | See ‘Data Collection and Samples’  Before conducting data analysis, we clean our data and excluded 113 invalid responses (e.g., questionnaires that didn’t pass the attention test, had missing values or too many duplicated answer). As such We only included 1087 completed questionnaires in our analysis. |
|  | Questionnaires submitted with an atypical timestamp | We have a set deadline for data collection, and the questionnaire filled in after this deadline was not reclaimed. |
|  | Statistical correction | N/A |
