# Supplementary material for: Impact of Concurrent Media Exposure on Professional Identity: Cross-Sectional Study of 1087 Medical Students During Long COVID
Source: J Med Internet Res. 2024 Oct 17;26:e50057. doi: 10.2196/50057 (PMC11528167; doi:10.2196/50057)
Supplement: Multimedia Appendix 2 [file jmir_v26i1e50057_app2.doc]

## Multimedia Appendix 2: Data Analysis Results of Pilot Test.

This study conducted a pilot test to assess the reliability and validity of the instruments with 63 medical students. The results are presented in Appendix 2-Table 1. Notably, Cronbach α, composite reliability values and average variance extracted (AVE) values for all the constructs were meeting the recommended threshold values of 0.7, 0.8 and 0.5 respectively [1,2]. Simultaneously all the item loadings exceeded 0.7. As such, the measurement is good in reliability and convergent validity. The discriminate validity analysis in Appendix 2-Table 2 show that the square roots of AVE exceed all the inter-construct correlation coefficients, which confirms that the current measurement has acceptable discriminant validity.

To avoid multicollinearity and common method bias (CMB) issues in the formal investigation, two further examinations were conducted. First, VIF values (in Appendix 2-Table 1) of all the variables were below 5, which is acceptable [3]. Therefore, the potential multicollinearity is absent. Second, the Harman’s single-factor test was conducted in SPSS Statistics 26.0 to assess CMB. The most substantial factor explained only 18.93% of the total variance, which was lower than the threshold value of 50% [3]. Besides, the highest correlation between constructs (in Appendix 2-Table 2) was 0.597 and lower than 0.900 [4]. As such, CMB was also not an issue in this investigation.

**Appendix 2-Table 1.** Reliability and convergent validity analysis.

| Item | | VIF^a^ | Item loading | Mean | SD | AVE^b^ | CR^c^ | α^d^ |
| --- | --- | --- | --- | --- | --- | --- | --- | --- |
| **Online news media exposure** | | | | | | | | |
|  | Online news media exposure 1 | 1.504 | 0.940 | 4.75 | 1.53 | 0.780 | 0.876 | .733 |
|  | Online news media exposure 2 | 1.504 | 0.822 | 3.52 | 1.61 |
| **Social media exposure** | | | | | | | | |
|  | Social media exposure 1 | 1.213 | 0.753 | 4.68 | 2.35 | 0.630 | 0.835 | .710 |
|  | Social media exposure 2 | 2.119 | 0.878 | 5.06 | 1.70 |
|  | Social media exposure 3 | 1.868 | 0.743 | 4.65 | 1.65 |
| **Informational support** | | | | | | | | |
|  | Informational support 1 | 1.306 | 0.723 | 5.62 | 1.22 | 0.640 | 0.842 | .719 |
|  | Informational support 2 | 1.568 | 0.852 | 5.33 | 1.20 |
|  | Informational support 3 | 1.465 | 0.820 | 5.29 | 1.53 |
| **Emotional support** | | | | | | | | |
|  | Emotional support 1 | 2.743 | 0.918 | 5.83 | 1.14 | 0.827 | 0.935 | .896 |
|  | Emotional support 2 | 3.245 | 0.910 | 5.71 | 1.30 |
|  | Emotional support 3 | 2.476 | 0.900 | 5.68 | 1.16 |
| **Belonging** | | | | | | | | |
|  | Belonging 1 | 1.537 | 0.815 | 5.43 | 1.60 | 0.736 | 0.893 | .820 |
|  | Belonging 2 | 2.189 | 0.874 | 5.78 | 1.22 |
|  | Belonging 3 | 2.222 | 0.884 | 5.71 | 1.20 |
| **Professional commitment** | | | | | | | | |
|  | Professional commitment 1 | 1.978 | 0.854 | 5.06 | 1.49 | 0.632 | 0.895 | .860 |
|  | Professional commitment 2 | 2.147 | 0.800 | 4.56 | 1.06 |
|  | Professional commitment 3 | 2.038 | 0.864 | 5.06 | 1.27 |
|  | Professional commitment 4 | 1.890 | 0.742 | 4.81 | 1.32 |
|  | Professional commitment 5 | 1.978 | 0.702 | 4.76 | 1.38 |

1. VIF: variance inflation factor.
2. AVE: average variance extracted.
3. CR: composite reliability.
4. α: Cronbach α

**Appendix 2-Table 2.** Discriminant validity analysis..

|  | Online news media  exposure | Social media  exposure | Informational support | Emotional support | Belonging | Professional commitment |
| --- | --- | --- | --- | --- | --- | --- |
| Online news media  exposure | *0.883*^a^ |  |  |  |  |  |
| Social media  exposure | 0.395 | *0.804*^a^ |  |  |  |  |
| Informational support | 0.321 | 0.241 | *0.800*^a^ |  |  |  |
| Emotional support | 0.169 | 0.215 | 0.621 | *0.909*^a^ |  |  |
| Belonging | 0.146 | 0.047 | 0.499 | 0.597 | *0.858*^a^ |  |
| Professional commitment | -0.026 | 0.065 | 0.315 | 0.307 | 0.429 | *0.795*^a^ |

1. The square roots of average variances extracted (AVEs) are in italic.

### Reference

1. Fornell C, Larcker DF. Structural Equation Models with Unobservable Variables and Measurement Error: Algebra and Statistics. Journal of Marketing Research 2018 Nov 28; 18(3):382-388 doi: [10.1177/002224378101800313](https://www.x-mol.com/paperRedirect/1357804485803028480)
2. Hair JF, Black WC, Babin BJ, Anderson RE. Multivariate data analysis: A global perspective. 7th ed. Upper Saddle River: Pearson Prentice Hall; 2010. ISBN: 0135153093
3. Harman HH. Modern factor analysis. Chicago: University of Chicago press; 1976. ISBN: 9780226316529
4. Bagozzi RP, Yi Y, Phillips LW. Assessing Construct Validity in Organizational Research. Administrative Science Quarterly 1991 Sept; 36(3):421 doi: 10.2307/2393203
